# Low Back Pain Among Weightlifting Adolescents and Young Adults

**DOI:** 10.7759/cureus.9127

**Published:** 2020-07-11

**Authors:** Mohamad Y Fares, Jawad Fares, Hamza A Salhab, Hussein H Khachfe, Ahmad Bdeir, Youssef Fares

**Affiliations:** 1 College of Medical, Veterinary and Life Sciences, University of Glasgow, Glasgow, GBR; 2 Neurological Surgery, Northwestern University Feinberg School of Medicine, Chicago, USA; 3 Surgery, American University of Beirut Medical Center, Beirut, LBN; 4 General Surgery, American University of Beirut Medical Center, Beirut, LBN; 5 Information Systems and Machine Learning Lab, Universität Hildesheim, Hildesheim, DEU; 6 Neuroscience Research Center, Lebanese University, Faculty of Medical Sciences, Beirut, LBN

**Keywords:** sports medicine, low back pain, weightlifting, back injury, spine rehabilitation, spine examination, lumbar spinal stenosis (lss), dorsal spine, physical medicine and rehabilitation

## Abstract

Background

Weightlifting is a common type of sports training that develops the strength and size of skeletal muscles. Low back pain (LBP) is one of its most common complaints. This sport has become prevalent among adolescents and young adults as they work to enhance their physique and body image. The aim of our study is to explore the nature and cause of LBP in weightlifting adolescents and young adults in an aim of extrapolating proper preventive measures.

Methods

Participants were patients who engaged in weightlifting sports and had presented to our clinic with nonspecific LBP. They were examined and asked to rate and localize their pain. Back positioning during weightlifting techniques along with other exercise habits was explored. Patients with congenital or systemic diseases and fractures were excluded from our study.

Results

A total of 93 patients presented with LBP (age range: 16-26 years), all of whom partook in weightlifting (N=93). Localized pain was found in 43 patients (46%). Pain radiating to the left side was found in 31 patients (33%), while pain radiating to the right was found in 19 patients (21%). LBP localized at the level of L4-L5 was found in 44 cases (47%), while that localized at L5-S1 was found in 43 cases (46%). Only six cases localized pain at the level of L3-L4 (7%). A total of 23 cases required surgery (25%), while others were managed conservatively. All the participants (100%) reported their pain to be initiated during or after weightlifting maneuvers. Psychological symptoms were found in 13 cases (19%). Factors that helped relieve the pain included surgery, swimming, and wearing a back brace.

Conclusion

Weightlifting is a sport that utilizes heavy weights to engage the muscles in the body, and consequently, predisposes athletes to LBP. Using excessive weights and performing improper techniques puts the back in a compromising position that may lead to injury. Medical and sports personnel should raise awareness on the biomechanical properties of the lumbar spine and the correct spine-protective posture during training to help prevent these injuries in the future.

## Introduction

Weightlifting is a sport that revolves around a regimen of exercises designed to improve the muscular development of the human body and endorse a healthy fit lifestyle [1]. It is also used to increase the size, shape, and symmetry of the individual’s superficial muscles so that they are larger, more noticeable, and better defined [2]. Barbells, dumbbells, and other resistance training devices are used in the exercises [1].

Weightlifting and other forms of strength training are becoming more popular due to an increased awareness of the need to maintain individual physical fitness [3]. When combined with proper aerobic fitness training, bodybuilding can often be healthy and beneficial. However, when the only objective is to obtain a better body image with no regards to proper techniques and procedures, the exercises may prove to be harmful [2]. As a result, bodybuilding injuries emerge and cause devastating outcomes to the individual.

Injuries to the lower back often arise in weightlifting due to improper execution of exercises and using excessive weights, and as a result, low back pain (LBP) is considered one of the most common complaints in the sport [4]. Adolescents and young adults are particularly vulnerable to this type of injury due to growth spurts, increased physical activity and increased engagement in sports [5]. LBP can pose prominent hardships to the patient by limiting movement, weakening strength, and eliciting painful sensations. In addition, LBP can pose a risk on the psychological wellbeing of the injured athlete, due to its chronicity and debilitating nature [6].

Obtaining a deeper knowledge with regards to the nature and cause of an injury is pivotal for establishing novel therapeutic modalities and proper prevention guidelines. Accordingly, this study aims to examine the characteristics of LBP in weightlifting sports in an attempt to extrapolate protective measures in the future.

## Materials and methods

This study explored cases of admitted to our clinic with nonspecific LBP. Demographic information, such as age, sex, weight, height, level of activity, and exercise habits and techniques, was collected from individuals and their parents.

Ages of interviewed individuals ranged between 16 and 26 years. All individuals partook in the sport of weightlifting and bodybuilding. Patients were asked to localize the pain on themselves and on a model provided to them. Neurological assessment was conducted to test for motor and sensory deficits. Patients were also asked about their pertinent exercise habits, weightlifting moves, and whether any exercises exacerbate the pain or relieve it. Further investigation explored whether the techniques used were implemented properly; participants were asked to show their usual back position when engaged in several weightlifting and fitness exercises. All interviewees underwent radiographic imaging to rule out scoliosis or any other pathology at the lumbar spine level. In addition, psychological and social symptoms were investigated by interviewing the patients and/or their parents. All patients were diagnosed and managed by the same spine surgeon.

For the purposes of this study, we did not include patients reporting pain associated with congenital or systemic diseases, such as scoliosis. We also excluded patients reporting pain resulting from frank injuries, such as fractures, and pain following surgical interventions.

## Results

A total of 93 patients, 87 men and six women, presented to our clinic with nonspecific LBP, all of which partook in weightlifting and fitness exercises. Ages of the patients ranged from 16 to 26 years with a mean age of 21 years. The 93 patients did not suffer from any systemic or congenital condition that caused their LBP, and were thus, the focus of our study (N=93) (Table 1).

**Table 1:** Distribution of patients according to sex, pain characteristics, surgery requirement, and vertebral level of lumbar pain.

	Sex		Pain Characteristics			Surgery Requirement		Lumbar Level		
	Male	Female	Localized	Radiating to the left	Radiating to the right	Yes	No	L3-L4	L4-L5	L5-S1
N	87	6	43	31	19	23	70	6	44	43
Percent (%)	94	6	46	34	20	25	75	7	47	46

**Figure 1:**
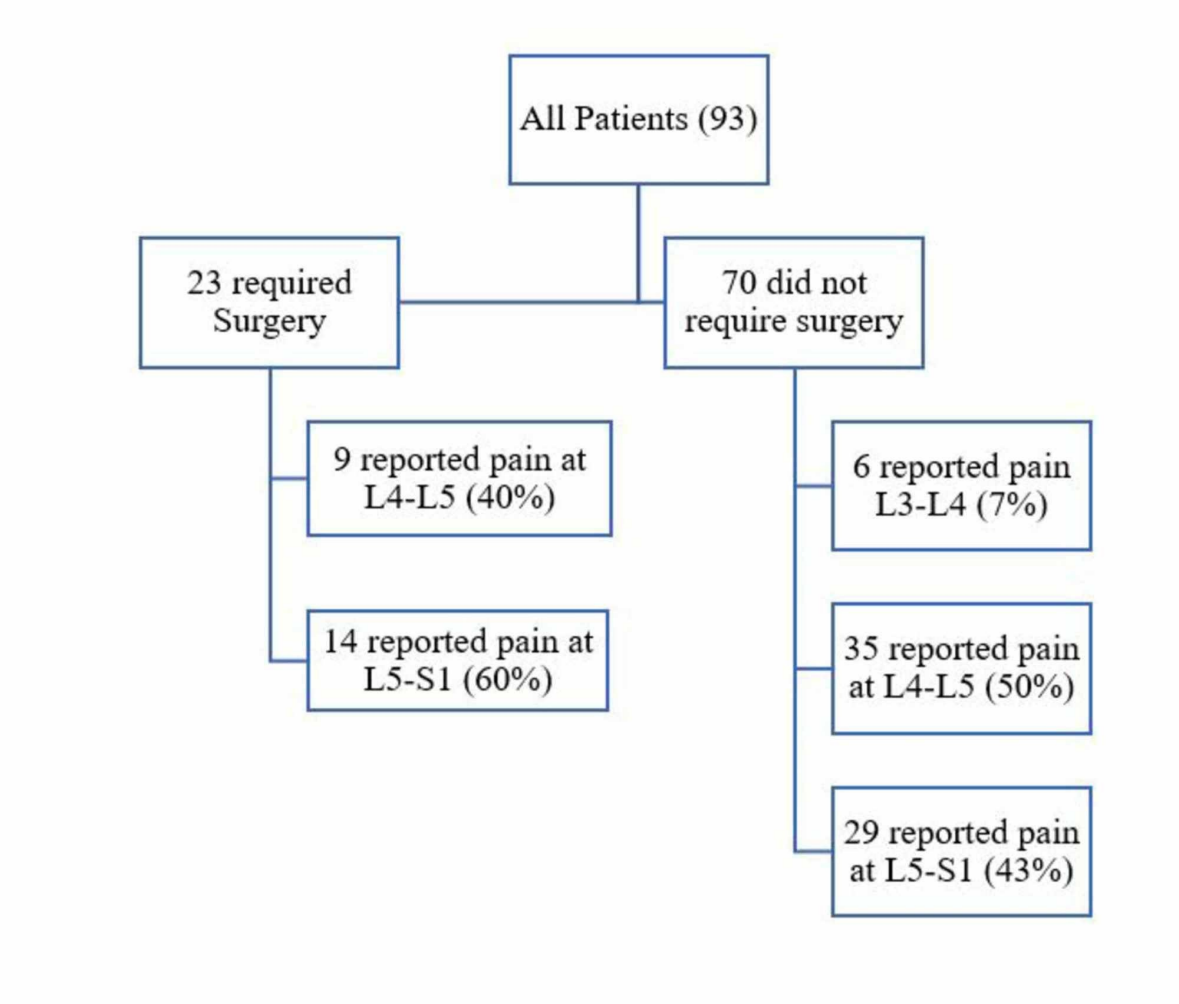
The affected vertebral segments in both surgical and nonsurgical low back pain patients.

Of the 93 patients, 43 reported localized LBP (46%), 31 reported LBP radiating to the left leg (33%), and 19 reported LBP radiating to the right leg (21%). Of the total number of cases, 23 required surgery (25%). LBP was mostly localized at the level of L4-L5 with 44 cases (47%) and L5-S1 with 43 cases (46%). Only six cases (7%) reported pain at the level of L3-L4 (Table 1). Of the cases that underwent surgery, 14 cases (60%) were operated at the level of L5-S1 and nine cases (40%) were operated at the level of L4-L5 (Figure 1).

Patients who did not undergo surgery underwent conservative treatment comprised of nonsteroidal anti-inflammatory medication (NSAIDs) and physical therapy for several years, with recurrent back pain. Recurrent pain caused psychological consequences on 13 of the nonsurgical cases (19%), mainly due to their inability to partake in sports activities. These patients reported feeling “hopeless” and “disabled”.

In all patients, reported LBP was caused by improper or flawed techniques adopted during weightlifting or fitness activities, especially during squats and deadlifts. Factors that helped relieve the pain included surgery, swimming, and wearing a back brace. Factors that exacerbated the pain included noncompliance with treatment regimen and a premature return to weightlifting exercises and sports.

## Discussion

LBP is a very common condition that impacts many sports and can affect any sex or age group; nevertheless, LBP is especially common in adolescents and young adults [5]. Studies suggest that the prevalence of LBP in this age category can be attributed to growth spurts, increased physical activity, and engagement in sports [5,7,8]. All of the patients in our study were adolescents or young adults who engaged in weightlifting and presented with LBP.

**Figure 2:**
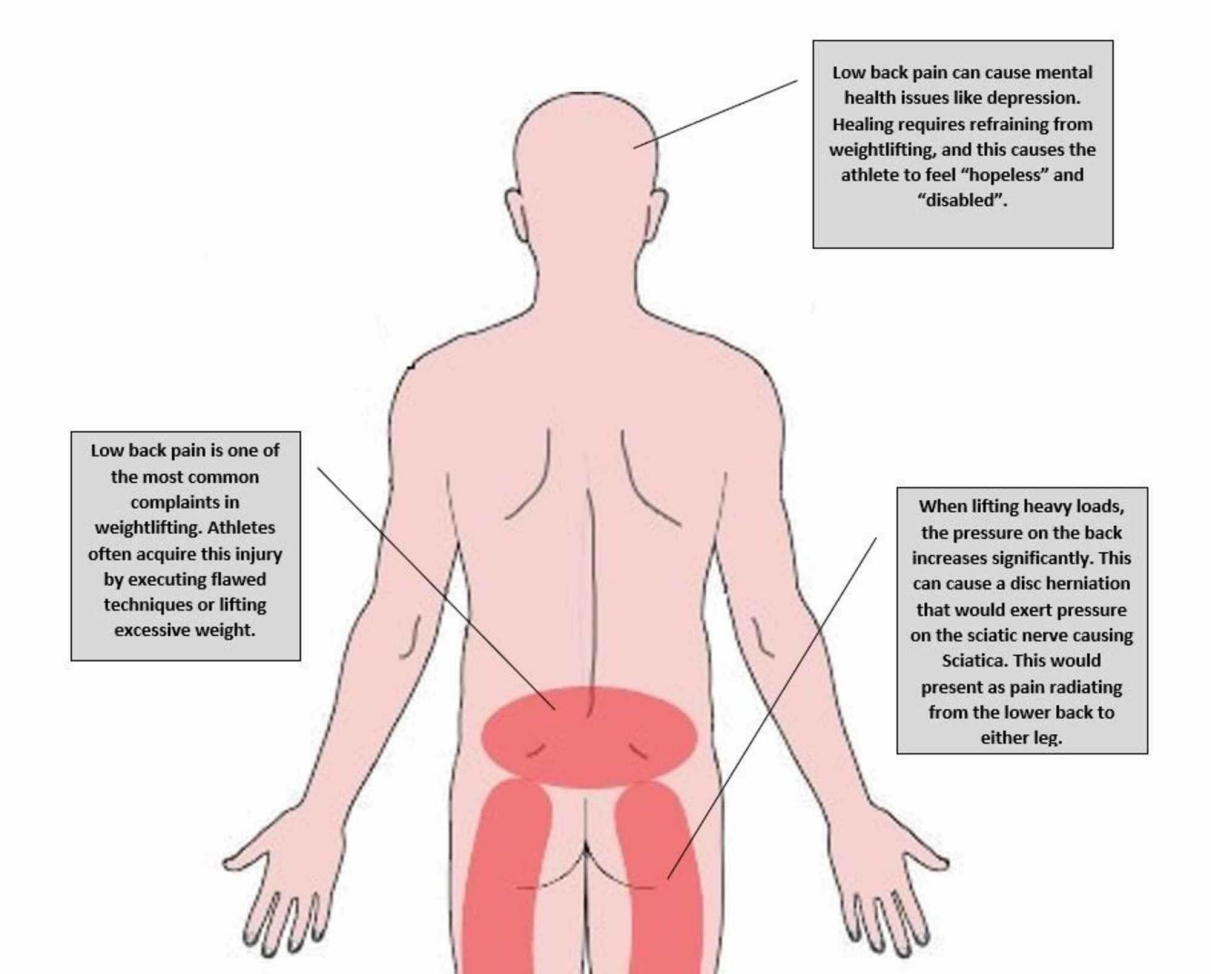
Low back pain in weightlifting athletes.

In the sport of weightlifting, LBP is considered one of the most common complaints with incidence rates reaching 40.8% [7,9]. This sport involves many defined moves and techniques that extensively engage the muscles in the back using heavy weights [10]. This affects the high joint moments, and increases the compressive loads and shearing forces on the spine and joints. As a result, injuries often occur due to improper execution of the techniques or lifting excessive weights [10-14]. Flawed techniques can often put a great deal of stress on the muscles of the lower back, and when the loads are too high, this causes muscle injury. The two most common injuries leading to LBP in this sport are muscle strains and intervertebral disc bulge or herniation [10]. While a muscle strain usually presents with a lengthy nonradiating LBP, a disc bulge or herniation causes LBP that may manifest with unilateral or bilateral radiation to the legs. Our study showed that 44% of injuries presented with localized pain, while 54% presented with radiating pain. This delineates the variation in the presentation of LBP in weightlifting (Figure 2).

**Figure 3:**
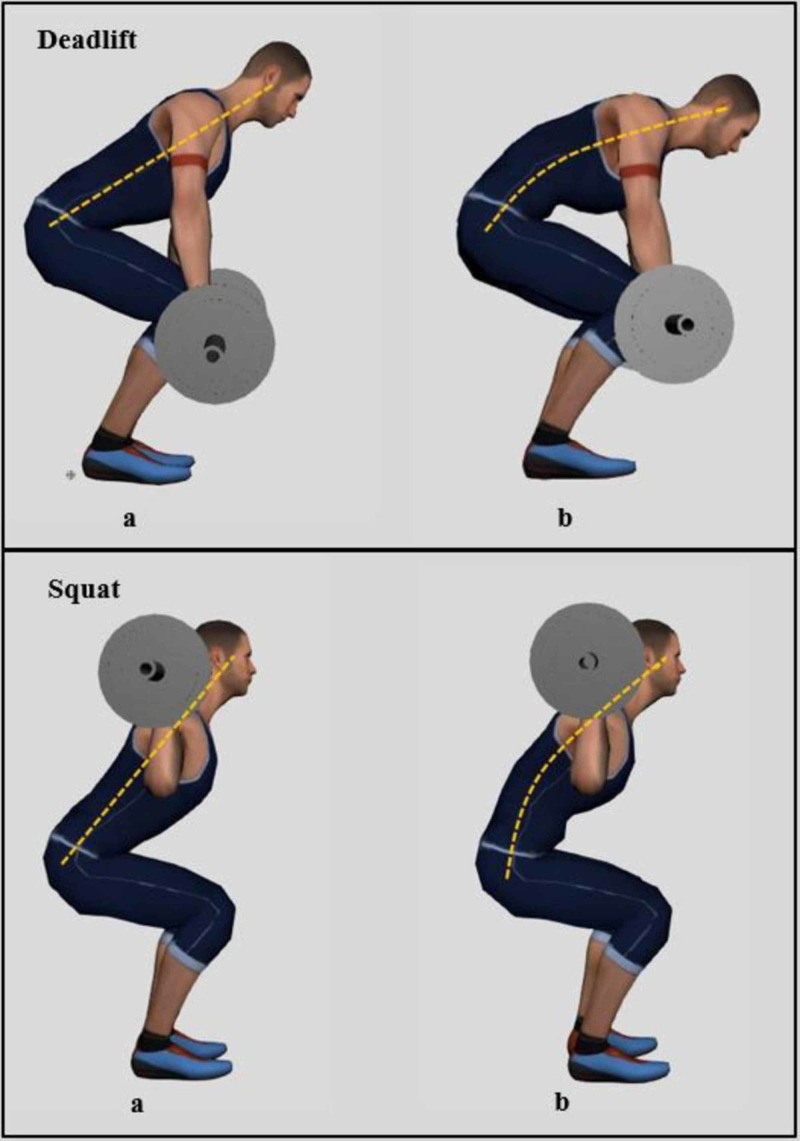
Figure showing the proper and improper execution of deadlifts and squats. During the deadlift, the athlete lifts the bar until legs get locked and the lifter’s posture is straight (Deadlift a), and during the squat, the athlete removes the bar from the rack and lowers the body until the hip joint gets lower than the knees (Squat a). Improper execution of these movements may compromise proper body posture and lead to body tilt and back flexion as seen in (Deadlift b and Squat b). As the body is tilted forward, the effects of the loads lifted increase dramatically and injury risk rises.

It has often been reported that body tilt and back flexion seen in many weightlifting techniques cause greater loads on the lumbar spine, damage the musculoskeletal system, and lead to pain [14]. Deadlifts and squats were reported to cause LBP more so than other exercises. During the deadlift, the athlete lifts the bar from the floor until legs get locked and the lifter’s posture is erect, and during the squat, the athlete removes the bar from the squat rack and lowers the body until the hip joint gets lower than the knees [10]. These two movements cause the extensors of the spine to oppose the muscular torque of the body in an attempt to prevent the body from collapsing with the load [10]. Improper execution of these movements may compromise proper body posture and lead to body tilt and back flexion (Figure 3). As the body is tilted forward, the effects of the loads lifted increase dramatically [15]. For example, when the body is tilted by 20 degrees, the compression forces on the fourth lumbar intervertebral disc increase by 50%. If we add 20 kilograms at this body position, these forces increase by 220%. One can then imagine the severe damage warranted to the lumbar region when the weightlifter’s posture is compromised beyond 20 degrees, and the weights carried exceed 20 kilograms. This renders the lumbar region highly prone to injuries that may vary in severity and presentation.

The variation in severity and presentation of LBP in weightlifting entails a subsequent variation in the modalities of treatment. While the majority of the patients in our study underwent conservative therapy, many cases necessitated surgical requirement. The primary method of intervention often included reassurance, analgesic medications, and physical therapy; in the cases where the pain persisted, surgical interventions were suggested [13]. Lumbar injuries are often debilitating and cause a decrease in the quality of life of the patient. Weightlifters with lumbar injuries are often advised to refrain from their sport for a long time in order to protect their lower back and avoid aggravation of their injury. This, in turn, takes a toll on the psychological well-being of the athlete and may cause mental health issues like depression, as seen with some of our patients [16]. Athletes often feel weak, hopeless, and disabled, and this can disrupt their work, social life, and/or education. As a result, it is often recommended to encourage the athlete to remain physically active as much as possible in a safe controlled setting. In mild cases, the weightlifter is advised to reduce the intensity of the lifting routine. It is also preferable to perform the more demanding and challenging part of the routine early in the training session to avoid technical errors due to fatigue and exhaustion [17]. Additional suggestions may include modifications in training regimens and usage of a protective back brace. In more severe cases, the athlete may be advised to lay off weightlifting and consider a different sport. Swimming, in particular, has been found to significantly reduce LBP and is often prescribed as a rehabilitation exercise for this condition [18]. The reason behind this is that swimming aids in relieving the pressure off the spine and helps strengthen the muscles of the back leading to a more stable and proper posture.

The absence of validated instruments to measure LBP is a limitation to similar studies [6]. This leads to a difficulty in drawing conclusions and conducting comparisons between populations and samples. Future studies should aim to find a unified validated instrument for measuring LBP in weightlifting, explore the relationships between LBP and other adverse health risk factors, and extrapolate preventive measures to help eradicate LBP from the setting of weightlifting sports.

## Conclusions

LBP is a very common condition that affects many people worldwide. Weightlifting is a popular sport that uses heavy weights to extensively engage the muscles in the body, and as a result, predispose athletes to LBP. Using excessive weights during weightlifting techniques can compromise body posture and lead to body tilt and back flexion. This increases the stress on the spine of the weightlifter and renders the lumbar area highly prone to injury. Proper knowledge on weightlifting techniques and close monitoring by physical specialists can help reduce lower back injuries in the weightlifting setting. Moreover, spine surgeons, physical therapists, and physical trainers should raise awareness on the biomechanical properties of the lumbar spine to help prevent these injuries in the future.

## References

[1] Dutton KR, Laura RS (1989). Towards a history of bodybuilding. Sporting Traditions.

[2] Kent M (2017). Food and Fitness: A Dictionary of Diet and Exercise. https://books.google.com.lb/books?hl=en&lr=&id=NaCPDgAAQBAJ&oi=fnd&pg=PT88&dq=Food+%26+Fitness:+A+Dictionary+of+Diet+%26+Exercise&ots=ywYQ2NyBNY&sig=bNrTyCQZ2fswj7acfmwsj6OZoEo&redir_esc=y#v=onepage&q=Food%20%26%20Fitness%3A%20A%20Dictionary%20of%20Diet%20%26%20Exercise&f=false.

[3] Busche K (2008). Neurologic disorders associated with weight lifting and bodybuilding. Neurol Clin.

[4] Vahdat I, Rostami M, Ghomsheh FT, Khorramymehr S, Tanbakoosaz A (2017). Effects of external loading on lumbar extension moment during squat lifting. Int J Occup Med Environ Health.

[5] Ganesan S, Acharya AS, Chauhan R, Acharya S (2017). Prevalence and risk factors for low back pain in 1,355 young adults: a cross-sectional study. Asian Spine J.

[6] Fares J, Fares MY, Fares Y (2017). Musculoskeletal neck pain in children and adolescents: risk factors and complications. Surg Neurol Int.

[7] Hestbaek L, Leboeuf-Yde C, Kyvik KO (2006). Are lifestyle-factors in adolescence predictors for adult low back pain? A cross-sectional and prospective study of young twins. BMC Musculoskelet Disord.

[8] Risser WL, Risser JM (1990). Weight-training injuries in adolescents. Am J Dis Child.

[9] Brown EW, Abani K (1985). Kinematics and kinetics of the dead lift in adolescent power lifters. Med Sci Sports Exerc.

[10] Siewe J, Rudat J, Röllinghoff M, Schlegel UJ, Eysel P, Michael JWP (2011). Injuries and overuse syndromes in powerlifting. Int J Sports Med.

[11] Cholewicki J, McGill SM (1992). Lumbar posterior ligament involvement during extremely heavy lifts estimated from fluoroscopic measurements. J Biomech.

[12] Cholewicki J, McGill SM, Norman RW (1991). Lumbar spine loads during the lifting of extremely heavy weights. Med Sci Sports Exerc.

[13] Maher C, Underwood M, Buchbinder R (2017). Non-specific low back pain. Lancet.

[14] Nowakowska K, Gzik M, Michnik R, Myśliwiec A, Jurkojć J, Suchoń S, Burkacki M (2017). The loads acting on lumbar spine during sitting down and standing up. Innovations in Biomedical Engineering.

[15] Jensen GM (1980). Biomechanics of the lumbar intervertebral disk: a review. Phys Ther.

[16] Lee J, Gupta S, Price C, Baranowski AP (2013). Low back and radicular pain: a pathway for care developed by the British Pain Society. Br J Anaesth.

[17] Caine DJ, Harmer PA, Schiff MA (2009). Epidemiology of Injury in Olympic Sports. https://books.google.com.lb/books?hl=en&lr=&id=JOWN_FNViMgC&oi=fnd&pg=PP2&dq=Epidemiology+of+injury+in+olympic+sports.&ots=ImBuYyzTlc&sig=zgyVpF4ff5oAkQZmJX24fKBAkVc&redir_esc=y#v=onepage&q=Epidemiology%20of%20injury%20in%20olympic%20sports.&f=false.

[18] Harreby M, Hesselsøe G, Kjer J, Neergaard K (1997). Low back pain and physical exercise in leisure time in 38-year-old men and women: a 25-year prospective cohort study of 640 school children. Eur Spine J.

